# Supplementary material for: Long-Term Results of Endovascular Treatment with Nitinol Stents for Femoropopliteal TASC II C and D Lesions
Source: Medicina (Kaunas). 2022 Sep 5;58(9):1225. doi: 10.3390/medicina58091225 (PMC9500608; doi:10.3390/medicina58091225)
Supplement: Supplementary file 1 [file medicina-58-01225-s001.zip › medicina-1864339-supplementary.pdf]

Table S1. Cox-regression analysis of factors associated with loss of secondary patency

Loss of Secondary Patency	Unadjusted HR (95% CI)	*p*-value	Adjusted* HR (95% CI)	*p*-value
TASC II D lesion	0.99 (0.53 – 1.84)	0.97		
Chronic total occlusion	1.74 (0.73 – 4.15)	0.21		
Lesion length > 25 cm	1.05 (0.56 – 1.96)	0.88		
Lesion length > 30 cm	0.98 (0.43 – 2.21)	0.73		
Previous intervention	0.94 (0.46 – 1.94)	0.87		
Reference vessel diameter < 5mm	0.58 (0.30 – 1.15)	0.12		
Severe calcification	0.77 (0.35 – 1.67)	0.51		
Stenosis free outflow vessels >1	0.53 (0.28 – 1.01)	0.05	0.51 (0.27 – 0.96)	0.04
Rutherford cat. 5 or 6	1.77 (0.84 – 3.72)	0.13		
No. of Stents > 4	1.09 (0.58 – 2.02)	0.66		
Stent diameter > 6mm	0.94 (0.50 – 1.78)	0.86		
Stent oversizing > 1mm	0.98 (0.51 – 1.86)	0.94		
Popliteal stenting	0.63 (0.30 – 1.32)	0.22		
Re-entry device used	0.59 (0.18 – 1.92)	0.38		
Trans-popliteal access	1.43 (0.73 – 2.82)	0.30		
Distal embolization	1.19 (0.37 – 3.86)	0.78		
> 1 local complication	1.74 (0.53 – 5.73)	0.36		

* Variables identified in univariate analysis ($p \leq .15$) were included in a multivariate Cox proportional hazards model for associations with loss of secondary patency.

Table S2. Complications.

	N=103, 109 lesions
Overall complications	45
Patients with ≥ 1 complication	7 (7)
Local complications	35
- Distal embolization	7
- Minor amputation	12
- Pseudo aneurysm	2
- Surgery for acute bleeding	4
- Vessel perforation	10
Systemic complications	10
- Anemia	5
- Congestive heart failure	2
- Renal function deterioration	3
